# Matrix Metalloproteinase-9 (MMP-9) as a Therapeutic Target: Insights into Molecular Pathways and Clinical Applications

**DOI:** 10.3390/pharmaceutics17111425

**Published:** 2025-11-04

**Authors:** Marta Wolosowicz, Slawomir Prokopiuk, Tomasz W. Kaminski

**Affiliations:** 1 Thrombosis and Hemostasis Program, VERSITI Blood Research Institute, Milwaukee, WI 53226, USA; 2 Faculty of Health Sciences, University of Lomza, 14 Akademicka St., 18-400 Łomża, Poland

**Keywords:** matrix metalloproteinase-9 (MMP-9), extracellular matrix (ECM) remodeling, disease-specific modulation, NF-κB/AP-1 signaling, cardiometabolic modulation, anti-inflammatory therapeutics, precision pharmacology

## Abstract

Matrix metalloproteinase-9 (MMP-9) is a zinc-dependent endopeptidase that plays a central role in extracellular matrix (ECM) remodeling, angiogenesis, immune cell trafficking, and cytokine activation. Dysregulated MMP-9 activity has been implicated in the pathogenesis of diverse conditions, including atherosclerosis, aneurysm formation, chronic obstructive pulmonary disease (COPD), asthma, neurodegeneration, and malignancy. Although broad-spectrum synthetic MMP inhibitors were initially developed as therapeutic agents, clinical trials failed due to lack of selectivity, poor tolerability, and impairment with physiological tissue repair. This outcome has shifted attention toward indirect pharmacological modulation of MMP-9 using drugs that are already approved for other indications. In this paper, we review the evidence supporting MMP-9 modulation by established therapeutics and adjunctive strategies. Cardiometabolic agents such as statins, angiotensin-converting enzyme (ACE) inhibitors, angiotensin II receptor blockers (ARBs), metformin, and pioglitazone reduce MMP-9 expression and enzymatic activity, contributing to vascular protection, improved insulin sensitivity, and attenuation of aneurysm progression. Anti-inflammatory and respiratory drugs, including glucocorticoids, phosphodiesterase-4 (PDE4) inhibitors, macrolide antibiotics, montelukast, and nonsteroidal anti-inflammatory drugs (NSAIDs), suppress MMP-9-driven airway inflammation and pathological tissue remodeling in asthma, COPD, and acute lung injury. Tetracycline derivatives, particularly sub-antimicrobial dose doxycycline, directly inhibit MMP-9 activity and are clinically validated in the treatment of periodontal disease and vascular remodeling. Hormone-related therapies such as rapamycin, estradiol, and tamoxifen exert tissue- and disease-specific effects on MMP-9 within endocrine and oncologic pathways. In parallel, nutritional interventions—most notably omega-3 polyunsaturated fatty acids and antioxidant vitamins—provide adjunctive strategies for mitigating MMP-9 activity in chronic inflammatory states. Taken together, these findings position MMP-9 as a modifiable and clinically relevant therapeutic target. The systematic integration of approved pharmacologic agents with lifestyle and nutritional interventions into disease-specific treatment paradigms may facilitate safer, context-specific modulation of MMP-9 activity and unveil novel opportunities for therapeutic repurposing.

## 1. Introduction

### 1.1. Dual Functions of MMP-9 in Tissue Homeostasis and Disease Progression

#### Structural and Functional Overview of MMP-9 in the ECM

Matrix metalloproteinase-9 (MMP-9), or gelatinase B, is a multi-domain zinc- and calcium-dependent endopeptidase whose structural organization directly determines its biochemical versatility and tight regulatory control. The N-terminal pro-domain contains the conserved *cysteine-switch* motif (PRCGVPD) in which a cysteine residue coordinates the catalytic zinc ion to maintain zymogen latency until proteolytic removal of the pro-peptide enables activation [1]. Crystallographic studies of the catalytic domain reveal that the active-site zinc is coordinated by three histidine residues (His401, His405, His411) with an essential Glu402 in the human enzyme. The catalytic domain also harbors a second structural zinc and multiple (at least five) calcium ions which stabilize the overall structural conformation and promote substrate recognition. Unique among the gelatinase subgroup, MMP-9 contains three fibronectin type II (FnII) repeats embedded within its catalytic domain; these modules form a collagen/gelatin-binding exosite that greatly enhances binding affinity for denatured collagen, elastin, and basement-membrane components and thereby increase proteolytic efficiency of large macromolecular substrates. A flexible O-glycosylated linker (hinge region) connects to the C-terminal hemopexin-like (PEX) domain, a ~ 200-residue four-bladed β-propeller structure that coordinates substrate specificity, homodimerization and natural inhibition via TIMP-1 docking [2,3]. Post-translational modifications (for example N-glycosylation at Asn38 and Asn120) further modulate secretion, stability and pericellular localization rather than catalytic rate per se. Collectively, this modular architecture couples precise enzymatic activation control with strategic substrate engagement and localization, positioning MMP-9 as a central effector of extracellular matrix (ECM) remodeling, cytokine and growth-factor release, barrier disruption and cell-migration dynamics. These structure-to-function relationships support both its essential physiological roles in development, angiogenesis and tissue repair and its pathological nature to drive maladaptive remodeling, chronic inflammation and metastatic dissemination when dysregulated [4,5].

Recent evidence further expands the concept of dual role of MMP-9 in health and disease [6,7,8]: Elevated serum MMP-9 levels are strongly associated with severe outcomes in acute inflammatory syndromes such as COVID-19 (with the significant weighted mean difference ~246 ng/mL in severe versus non-severe cases), indicating that MMP-9 upregulation parallels systemic proteolytic burden and acute tissue injury [9]. In chronic lung disease, patients with COPD display significantly higher serum MMP-9 and elevated MMP-9/TIMP-1 ratios, which correlate negatively with lung-function parameters such as FEV_1_ and FEV_1_/FVC, underlying the role of MMP-9 in airway remodeling and functional decline [10]. In the vascular biology, a 2024 Mendelian-randomization analysis has implicated MMP-9 expression as causally linked to thoracic and abdominal aortic aneurysm risk, thereby linking proteolytic imbalance to structural vascular failure [11]. In fibrotic disorders, MMP-9 is pivotal player in progressing fibrosis across organs, not only by degrading ECM but also by releasing profibrotic mediators and enabling fibroblast migration, thereby converting repair into maladaptive scarring [12]. Taken together, these findings clarify our understanding of MMP-9’s pathologic significance: it is not just a marker of matrix turnover, but a mechanistic effector in acute injury, chronic remodeling, barrier compromise, and progressive organ dysfunction. 

### 1.2. The Role of MMP-9 Angiogenesis and Vascular Remodeling

Angiogenic regulation represents another critical dimension of MMP-9 function. Through proteolytic degradation of basement membranes and interstitial ECM components, MMP-9 releases matrix-sequestered growth factors, including vascular endothelial growth factor (VEGF) and fibroblast growth factor (FGF), thereby amplifying local proangiogenic signaling and sustaining tumor vascularization and progression [13]. Experimental evidence demonstrates that MMP-9 deficiency compromises angiogenic responses, whereas its overexpression enhances neovascularization under both physiological and pathological conditions. Beyond mobilizing angiogenic growth factors, MMP-9 also generates bioactive ECM fragments, such as endostatin, that can exert tissue-dependent pro- or anti-angiogenic effects. This dualistic activity reflects the tightly controlled regulation of MMP-9 during angiogenesis, where transient activation facilitates tissue repair, while persistent overexpression drives pathological vascular remodeling in cancer, retinopathy, and chronic inflammatory disorders [14].

### 1.3. MMP-9 in Leukocyte Trafficking and Immune Regulation

Immune cell transmigration critically depends on the proteolytic activity of MMP-9. During inflammation, leukocytes must breach the basement membrane and interstitial ECM; by cleaving type IV collagen, laminin, and other basal membrane components, MMP-9 forms localized biotracks that facilitate neutrophil and macrophage extravasation [15]. Beyond degrading structural barriers, MMP-9 shapes the inflammatory *milieu* by processing chemokines such as CXCL8/interleukin-8 (IL-8) and cytokines including tumor necrosis factor-α (TNF-α), thereby enhancing leukocyte recruitment and amplifying inflammatory signaling [16]. Experimental studies reveal that MMP-9-deficient mice display markedly reduced neutrophil infiltration into inflamed tissues, underscoring its non-redundant role in innate immunity. However, while MMP-9 activity is indispensable for effective host defense, its excessive or sustained expression drives chronic inflammatory pathology by perpetuating leukocyte infiltration and tissue injury, as seen in rheumatoid arthritis, inflammatory bowel disease, and chronic pulmonary disorders [17]. Thus, MMP-9 functions as both a gatekeeper, enabling leukocyte passage across extracellular barriers, and an amplifier, intensifying immune cell trafficking: outcomes governed by the magnitude and temporal regulation of its activity.

### 1.4. Cytokine and Chemokine Processing by MMP-9

Beyond structural remodeling of the ECM, MMP-9 exerts critical immunomodulatory functions through the proteolytic processing of cytokines and chemokines. By cleaving and modifying these signaling mediators, MMP-9 alters their bioavailability, receptor affinity, and downstream biological effects [18,19]. For instance, proteolytic activation of pro-tumor necrosis factor-α (pro-TNF-α) enhances its pro-inflammatory potency, whereas MMP-9-mediated truncation of IL-8/CXCL8 generates N-terminally clipped isoforms with increased neutrophil chemotactic activity. Similarly, MMP-9 processing of chemokines such as CXCL5 and CXCL12 can either amplify or attenuate leukocyte recruitment, depending on the cellular and tissue microenvironment [20,21]. These post-translational modifications constitute an additional regulatory layer of immune control that cannot be achieved through transcriptional regulation alone. By proteolytically regulating cytokine and chemokine activity, MMP-9 functions as a molecular modulator, calibrating both the amplitude and duration of inflammatory cascades. When this regulation is lost, the balance shifts toward pathological inflammation, as seen in autoimmune diseases, chronic obstructive pulmonary disease, and sepsis—conditions in which excessive chemokine activation drives excessive leukocyte infiltration and collateral tissue damage [22]. Conversely, insufficient MMP-9 activity can impair inflammatory resolution and impair tissue repair. This bidirectional role underscores why emerging therapeutic strategies increasingly emphasize state-specific modulation of MMP-9 activity rather than indiscriminate inhibition [23].

### 1.5. Growth Factor Activation and Fibrotic Remodeling Mediated by MMP-9

A central mechanism by which MMP-9 governs tissue responses beyond ECM remodeling involves the proteolytic activation of growth factors such as transforming growth factor-β (TGF-β), VEGF, fibroblast growth factors (FGFs), and platelet-derived growth factor (PDGF), which are normally sequestered within the ECM in latent or binding-protein-associated forms [24]. Through cleavage of matrix scaffolds and carrier proteins, MMP-9 releases these immobilized factors into the pericellular *milieu*, increasing their bioavailability and receptor engagement. This activity promotes fibroblast activation, myofibroblast differentiation, and matrix protein deposition—hallmarks of fibrotic remodeling in the lung, kidney, and cardiovascular system [25]. In vascular tissues, MMP-9-mediated release of VEGF and PDGF stimulates smooth muscle cell proliferation and vessel wall remodeling—responses that are beneficial during repair but pathogenic in aneurysm expansion and atherosclerotic plaque destabilization [26,27]. Within the tumor microenvironment, MMP-9-dependent release of matrix-bound growth factors amplifies proangiogenic signaling, activates stromal fibroblasts, and drives epithelial–mesenchymal transition (EMT), thereby promoting neovascularization, invasive growth, and metastatic dissemination [28]. Collectively, these findings identify MMP-9 as a pivotal regulator of growth factor bioavailability, linking extracellular proteolysis to fibrotic, vascular, and neoplastic remodeling [23,24,25,26,27,28,29].

### 1.6. MMP-9 as a Biomarker and Therapeutic Target in Neurological Disease

Disruption of the blood–brain barrier (BBB) is a hallmark of numerous acute and chronic neurological disorders, with MMP-9 acting as a central effector. By proteolytically degrading tight junction proteins, including claudin-5, occludin, and zonula occludens-1 (ZO-1), MMP-9 compromises endothelial integrity and induces pathological increases in paracellular permeability [30]. This enzymatic activity primes the unregulated influx of plasma proteins, leukocytes, and neurotoxic molecules into the brain parenchyma, amplifying neuroinflammation and vasogenic edema. In experimental models of ischemic stroke, traumatic brain injury, and multiple sclerosis, early surges in MMP-9 correlate closely with BBB breakdown and worsened neurological outcomes [31]. Beyond junctional degradation, MMP-9 destabilizes the neurovascular interface by cleaving basement membrane components and releasing matrix-bound mediators that sustain endothelial dysfunction [26]. Elevated MMP-9 levels in cerebrospinal fluid and plasma are consistently associated with hemorrhagic transformation following thrombolytic therapy in stroke, highlighting its dual significance as both a mechanistic driver of vascular injury and a clinically relevant biomarker [32]. In neurodegenerative diseases such as Alzheimer’s and Parkinson’s, persistent MMP-9 activity induces low-grade BBB leakage, facilitating accumulation of inflammatory mediators and neurotoxic proteins that exacerbate neuronal damage and accelerate disease progression [33]. Importantly, experimental inhibition of MMP-9—through tetracyclines, statins, or genetic deletion—reduces BBB permeability, limits cerebral edema, and improves survival in animal models, emphasizing its therapeutic relevance. However, because transient BBB permeability may also facilitate immunosurveillance and repair, state-specific modulation rather than non-selective inhibition of MMP-9 is increasingly recognized as the most promising therapeutic approach [34,35].

### 1.7. MMP-9 in Tumor Progression and Metastasis

MMP-9 has been extensively studied in oncology, representing one of the most comprehensively investigated areas of its biology. MMP-9 promotes cancer progression through basement-membrane degradation, induction of epithelial–mesenchymal transition, and growth-factor-driven angiogenesis, collectively enabling invasion, dissemination, and metastatic expansion. Experimental models consistently show that MMP-9 overexpression accelerates tumor progression, whereas genetic deletion or pharmacologic inhibition suppresses invasion and reduces metastatic burden [36]. Clinically, elevated MMP-9 levels, whether in tumor tissue or circulation, correlate with poor prognosis, increased metastatic potential, and therapeutic resistance across malignancies including breast, colorectal, lung, and prostate cancers [37]. Because MMP-9 activity integrates ECM remodeling, growth-factor activation, and immune modulation, its role in malignancy is inherently multifaceted and disease-specific. This functional complexity underlies why MMP-9 remains a compelling yet technically challenging therapeutic target in cancer biology [37,38].

### 1.8. MMP-9 in Fibrosis and Chronic Inflammation

**Figure 1:**
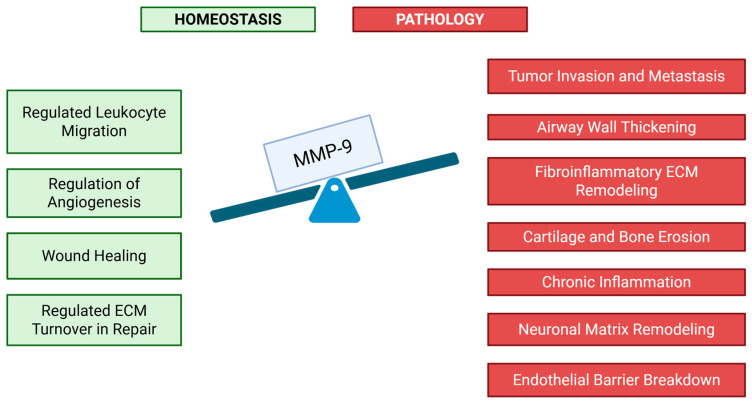
Dual Roles of MMP-9 in Health and Disease. Created in BioRender. Kaminski, T. (2025) https://BioRender.com/zavbi93.

Fibrosis and chronic inflammation exemplify disease momentum in which sustained MMP-9 activity transitions from adaptive to pathogenic. Under physiological conditions, MMP-9 facilitates matrix turnover during wound healing and resolution of acute injury [39]. Persistent upregulation, often driven by ongoing immune activation or oxidative/metabolic stress, disrupts the equilibrium between matrix degradation and deposition. In pulmonary fibrosis, elevated MMP-9 fosters aberrant fibroblast activation and disordered alveolar remodeling, correlating with disease progression and loss of lung function [40]. In rheumatoid arthritis, excessive MMP-9 released from synovial fibroblasts and infiltrating neutrophils accelerates cartilage erosion and promotes progressive joint destruction. Within the cardiovascular system, chronic MMP-9 activity compromises vascular integrity by weakening extracellular scaffolds and inducing fibro-inflammatory remodeling of vessel walls. This process increases atherosclerotic plaque vulnerability and contributes to maladaptive left-ventricular remodeling after myocardial infarction [41,42]. Moreover, in chronic disease states, MMP-9 generates bioactive ECM fragments—matrikines—that perpetuate inflammation and fibrosis through reciprocal immune activation. Whereas transient MMP-9 activity supports effective tissue repair, its persistent overexpression establishes a self-reinforcing cycle of inflammation and maladaptive remodeling that culminates in progressive organ dysfunction. This functional duality underscores the therapeutic potential of selective, state-specific modulation of MMP-9 aimed at suppressing chronic activity while preserving its reparative functions [2] (Figure 1).

### 1.9. Additional Mechanistic Links to MMP-9 Function: PON1, Endothelial Function, and Hyperhomocysteinemia

Paraoxonase-1 (PON1), an HDL-bound lactonase with potent anti-oxidative and anti-inflammatory activity, acts upstream of signaling pathways that transcriptionally control MMP-9 [43]; when PON1 activity decreases (either due to HDL dysfunction during inflammation or reduced PON1 secretion), redox tone and pro-inflammatory signaling increase, enhancing NF-κB/AP-1–dependent MMP-9 transcription and tipping tissues toward matrix injury and barrier failure [44]. Mechanistically, recent reviews and experimental studies show that PON1 mitigates oxidized-lipid stress and suppresses innate immune activation in monocyte-macrophage and endothelial compartment-functions tightly linked to limiting NF-κB/AP-1 activity, the canonical drivers of the MMP-9 promoter. Thus, reduced PON1 is expected to de-inhibit MMP-9 expression and gelatinolysis in inflamed vascular beds and tissues. Consistent with this axis, human cohort data in untreated familial hypercholesterolemia demonstrate network correlations among PON1 arylesterase activity, MMP-9, and inflammatory mediators (sCD40L, S1P), positioning PON1 within an HDL-inflammation-protease module relevant to endothelial dysfunction. In parallel, disease pathologies characterized by mTOR-driven suppression of PON1 glycosylation/secretion show immune dysregulation that would be permissive for protease induction, reinforcing the plausibility that PON1 loss removes a brake on MMP-9-mediated ECM remodeling [45]. Finally, multiple recent studies confirm that NF-κB/AP-1 signaling directly controls MMP-9 transcription under inflammatory stimuli (e.g., TNF-α, hyperglycemia), providing a clear mechanistic approach through which reduced PON1 activity can propagate to MMP-9 overexpression and disease progression [46,47,48]. Together, these lines of evidence support a model in which PON1 preserves HDL’s anti-inflammatory competence and restrains MMP-9; when PON1 is low or dysfunctional, the ensuing oxidative/inflammatory milieu unlocks MMP-9 transcriptional programs, accelerating matrix degradation, barrier leak, and chronic tissue injury.

MMP-9 links inflammatory signaling to endothelial dysfunction by targeting all three structural tiers that maintain barrier homeostasis, namely: glycocalyx, tight junctions, and basement membrane. At the luminal interface, MMP-9-mediated proteolysis accelerates endothelial glycocalyx degradation, impairing mechanosensory and anti-thromboinflammatory functions while exposing underlying adhesion molecules that promote leukocyte–endothelial interactions; in patients with hemorrhagic fever with renal syndrome, circulating MMP-9 levels correlated with markers of glycocalyx degradation and organ injury, and mechanistic study identified MMP-9 as a mediator of eGLX shedding in vitro and in vivo [49]. At intercellular junctions, MMP-9 directly compromises tight-junction complexes (e.g., claudin-5, occludin, ZO-1), increasing paracellular permeability and promoting blood–tissue barrier disruption; in the cerebral endothelium, VEGF-MMP-9 signaling compromises tight junctions and disrupts transport systems, and selective MMP-9 blockade attenuates blood–brain barrier breakdown in stroke models [50]. At the abluminal surface, MMP-9 degrades basement-membrane scaffolds (collagen IV, laminin), debilitating endothelial anchorage and facilitating leukocyte diapedesis, edema, and capillary leak—core features of endothelial dysfunction, which were summarized across recent reviews [51]. Clinically, symptoms associated with acute vascular stress show consistent MMP-9 and markers of barrier injury elevation: during cardiopulmonary bypass, perioperative peaks in serum MMP-9 paralleled shedding of glycocalyx components, implicating protease-driven barrier compromise [52]. Upstream, canonical inflammatory pathways (TNF-α, ROS, macrophage-derived mediators) induce MMP-9 via NF-κB/AP-1 and related pathways, establishing a feed-forward loop in which proteolysis amplifies leukocyte recruitment and oxidative stress, further depressing nitric-oxide bioavailability and vascular reactivity—a hallmark of endothelial dysfunction [53,54,55]. Together, these data position MMP-9 not as a bystander marker but as an executable node that integrates inflammatory activation with structural barrier impairment; then also rationalize therapeutic strategies that pair endothelial-protective agents with precise MMP-9 inhibition to restore vascular integrity in inflammatory and ischemic disease states.

Elevated homocysteine (Hcy) exerts a pro-inflammatory and pro-remodeling stimulus that converges on MMP-9 upregulation and activation, thereby bridging metabolic dysregulation and ECM perturbation in inflammatory disease states. Mechanistically, Hcy induces reactive oxygen species (ROS) and triggers a pertussis-toxin sensitive G-protein-coupled receptor (GPCR) cascade, initiating intracellular Ca^2+^ fluxes, protein tyrosine kinase (PTK) and protein kinase C (PKC) activation which then leads to phosphorylation of the ERK1/2 MAPK axis in endothelial and other vascular cells; this pathway culminates in enhanced MMP-9 transcription, zymogen activation and gelatinolytic activity [56,57]. Once released, MMP-9 degrades basement-membrane and interstitial ECM components, disrupts endothelial and epithelial barrier integrity (e.g., increased paracellular permeability), and liberates ECM-sequestered cytokines and chemokines, thereby amplifying leukocyte infiltration, vascular leak and chronic inflammatory signaling. In conditions of hyper-homocysteinaemia (HHcy), such as in diabetes, vascular disease or certain inflammatory disorders, Hcy-driven MMP-9 activity promotes a feed-forward loop: ECM disruption strengthens inflammatory cell activity, inflammation sustains Hcy-mediated cell stress and MMP-9 induction, and barrier damage accelerates organ dysfunction. For example, in retinal endothelial cells Hcy increased MMP-9 expression and permeability changes consistent with microvascular injury [58]. Although direct clinical cohort studies remain relatively limited, elevated serum Hcy correlates with higher MMP-9 levels in human inflammatory conditions (e.g., epilepsy, vascular aneurysm) suggesting translational relevance [59]. Taken together, this mechanistic axis of Hcy-ERK1/2/PKC-MMP-9-ECM barrier disruption offers a plausible biochemical pathway linking metabolic perturbation to matrix remodeling and inflammatory disease progression.

In summary, the biological impact of MMP-9 is profoundly state/tissue-dependent, differing sharply between acute and chronic settings. During acute injury or infection, transient MMP-9 release from neutrophils and macrophages facilitates leukocyte recruitment, pathogen clearance, and re-epithelialization, thereby accelerating tissue repair [60]. In wound healing, timely MMP-9 activity supports keratinocyte migration and angiogenic remodeling—processes essential for restoration of barrier integrity. In contrast, persistent MMP-9 expression beyond the acute phase converts reparative proteolysis into pathology [61]. Chronic MMP-9 activity promotes aneurysm expansion by degrading elastin and weakening vascular walls, while in neurodegenerative diseases, sustained proteolysis disrupts the blood–brain barrier and exacerbates neuronal injury. This dichotomy underscores the need for therapeutic strategies focused on temporal precision rather than non-selective inhibition.

The present review synthesizes current evidence on how clinically approved drugs and investigational agents modulate MMP-9 expression and activity, aiming to point out therapeutic opportunities for state-specific regulation across inflammatory, fibrotic, vascular, and neurodegenerative diseases.

## 2. Druggability and Translational Potential of MMP-9

Given its central role in coordinating inflammation and ECM remodeling, dysregulated MMP-9 activity directly contributes to tissue injury and disease progression, making it a rational and tractable therapeutic target. Because MMP-9 is secreted and functions in the extracellular *milieu*, it is inherently more “druggable” than intracellular kinases or transcription factors. Its catalytic pocket and auxiliary domains are physically accessible to diverse therapeutic modalities—including small-molecule zinc chelators, domain-selective inhibitors, engineered tissue inhibitors of metalloproteinases (MMPs), and monoclonal antibodies—and its enzymatic activity can be monitored in biofluids such as plasma or cerebrospinal fluid (CSF) to guide dosing and response. This extracellular localization also permits localized delivery (e.g., topical, inhaled, or intralesional), minimizing systemic exposure and off-target toxicities: a principle increasingly recognized across extracellular protease biology and reinforced for MMP-9 in recent translational analyses [62,63,64]. Elevated MMP-9 concentrations in circulation or within diseased tissues consistently correlate with greater disease severity, poorer prognosis, and adverse clinical outcomes, reinforcing its dual value as both a biomarker and a therapeutic anchor. Collectively, these correlations provide clinical validation that MMP-9 modulation is not only mechanistically plausible but also poised to exert tangible impact on disease trajectories [65,66].

### 2.1. Lessons from Broad-Spectrum Metalloproteinase Inhibition

Building on this rationale, early therapeutic programs aimed to directly suppress MMP-9 catalytic activity through broad-spectrum metalloproteinase inhibition—an approach that showed preclinical promise but ultimately proved very limited in the clinic. First-generation hydroxamate inhibitors, including batimastat (BB-94) and marimastat (BB-2516), advanced to Phase II/III oncology trials and exploratory studies in arthritis but yielded minimal survival or clinical benefit [67]. Their development was further devastated by a dose-limiting musculoskeletal syndrome (MSS) characterized by arthralgia, myalgia, stiffness, tendinopathy, and contracture, typically emerging after 1 to 3 months of therapy and resolving upon dose reduction or discontinuation. Randomized trials of marimastat in pancreatic cancer failed to demonstrate efficacy versus gemcitabine, alone or in combination, while frequently reporting MSS-related adverse events. Subsequent analyses implicated on-target and off-target inhibition of multiple MMP isoforms as the principal cause of these toxicities and the broader clinical failure of non-selective agents. These experiences catalyzed a paradigm shift toward isoform- and domain-selective MMP inhibition strategies designed to preserve efficacy while improving tolerability [68,69].

### 2.2. Toward Selectivity and Temporal Precision

MMP-9 is not uniformly deleterious. As stated before, during acute injury and early repair, regulated gelatinase activity supports ECM remodeling, angiogenesis, leukocyte trafficking, and re-epithelialization. Broad or prolonged inhibition at these stages can disrupt normal healing and worsen outcomes. This context dependence, reiterated in recent wound-healing and inflammatory-disease literature, underscores that effective interventions must be phase-specific: for example, transient suppression during chronic overexpression or pathologic remodeling, but not during early reparative windows [70,71]. Foundational clinical pharmacology revealed key developability gaps. Batimastat exhibited poor oral bioavailability and solubility, necessitating parenteral administration with local tolerability issues. Marimastat, while orally available, demonstrated variable systemic exposure and dose-limiting musculoskeletal toxicity, constraining efficacious dosing in oncology and arthritis trials. Together, these pharmacokinetic and toxicity liabilities narrowed the therapeutic index and yielded neutral or unfavorable risk–benefit outcomes [72].

In sub-summary, the triad of non-selective target engagement, biological phase mismatch with MMP-9 reparative roles, and suboptimal pharmacokinetic/toxicity profiles explains the underperformance of early broad-spectrum metalloproteinase inhibitors. These insights redirected the field toward precision inhibition: emphasizing isoform and domain selectivity, exosite binding, and antibody or TIMP-mimetic designs, coupled with case-dependent dosing and improved deliverability (local, topical, inhaled, or depot formulations) to restrain pathological MMP-9 activity while preserving its physiological functions.

## 3. Indirect and Signal-Aware Modulation of MMP-9

### MMP-9 as a Target for Precision Modulation

Given the clinical limitations of broad-spectrum metalloproteinase inhibition, current strategies increasingly focus on indirect modulation of MMP-9 through repurposed or pleiotropic agents that attenuate its expression or activity without abolishing physiological signaling. Compounds such as statins, tetracyclines (e.g., sub-antimicrobial dose doxycycline), angiotensin II receptor blockers (ARBs; e.g., losartan), macrolides (e.g., azithromycin or clarithromycin), glucocorticoids, peroxisome proliferator-activated receptor (PPAR) agonists, and select polyphenols converge on upstream regulatory nodes—including NF-κB, AP-1, MAPK/ERK, Toll-like receptor (TLR) pathways, and oxidative-stress signaling—to suppress MMP-9 transcription, limit neutrophil degranulation, enhance tissue inhibitor of metalloproteinase expression, and reduce protease release from macrophages and endothelial cells. This signal-specific attenuation preserves MMP-9 transient, reparative roles in early injury while dampening the chronic proteolytic tone that drives vascular leak, barrier failure, and maladaptive remodeling. From a translational standpoint, these agents offer favorable safety profiles, oral bioavailability, and dosing flexibility, enabling phase-specific and combination approaches: for example, pairing anti-inflammatory pathway control with domain-selective MMP-9 inhibition to enhance therapeutic precision and expand the druggable landscape [73,74].

This emerging therapeutic paradigm reframes MMP-9 not as a target to silence, but as one to recalibrate. The prevailing consensus emphasizes context-specific, partial modulation—suppressing the chronic, tissue-damaging proteolytic tone while preserving the transient physiological functions required for repair, angiogenesis, and immune resolution. This precision-inhibition concept has redirected drug-development efforts toward agents that precisely adjust MMP-9 indirectly, acting through upstream regulatory pathways rather than direct catalytic blockade. Within this framework, repurposed pharmacologic classes, including statins, tetracyclines, ARBs, and macrolides, have gained renewed interest for their capacity to attenuate MMP-9 expression or release in disease-specific conditions while maintaining favorable safety margins. The following sections summarize how these agent classes achieve such modulation, detailing their mechanistic pathways, supporting evidence, and therapeutic implications across vascular, inflammatory, and fibrotic pathologies.

## 4. Cardiometabolic Modulators of MMP-9 Activity

### 4.1. Statins: Indirect Modulators of MMP-9 via Mevalonate Pathway Interference

Beyond their canonical lipid-lowering activity via inhibition of 3-hydroxy-3-methylglutaryl-coenzyme A (HMG-CoA) reductase, statins (e.g., atorvastatin, simvastatin, rosuvastatin, fluvastatin) exert pleiotropic effects on inflammatory and proteolytic signaling, notably suppressing circulating and tissue MMP-9 levels [74]. This downregulation occurs in part through inhibition of isoprenoid synthesis—specifically farnesyl pyrophosphate and geranylgeranyl pyrophosphate—thereby preventing prenylation of small GTPases such as RhoA and Rac1 that activate NF-κB, MAPK/ERK, and AP-1 transcriptional pathways governing MMP-9 expression and secretion. Experimental data demonstrate that statin treatment reduces MMP-9 release from macrophages and vascular cells, attenuates TGF-β-induced MMP-9 expression in vitro, and suppresses MMP-9 levels in animal and patient models of vascular inflammation and fibrosis. For instance, Kim et al. showed that statins inhibit TGF-β_2_-stimulated MMP-9 and MMP-2 induction by interfering with mevalonate pathway intermediates [73], while Sheridan et al. summarized multiple studies reporting reduced MMP-9 secretion and activity in macrophage and vascular systems. Despite these mechanistic insights, meta-analyses of randomized trials indicate that plasma MMP-9 reduction is modest or inconsistent, suggesting that tissue-level modulation and pathology-specific effects may be more therapeutically relevant [75,76]. Clinically, statin therapy has been associated with lower serum and tissue MMP-9 concentrations in patients with atherosclerosis and post-revascularization, correlating with improved plaque stability and reduced rupture risk. In particular, rosuvastatin dose-dependently suppressed plasma MMP-9 and MMP-2 levels in patients with vascular disease, supporting a mechanism for statin-mediated plaque stabilization [77]. Statins also downregulate endothelial MMP-9 expression by inhibiting cyclooxygenase-2 (COX-2) and downstream inflammatory signaling [78].

### 4.2. ACE Inhibition: Indirect Recalibration of MMP-9 via RAS Blockade

Angiotensin-converting enzyme (ACE) inhibitors such as perindopril and captopril attenuate MMP-9 expression by suppressing angiotensin II-dependent oxidative and inflammatory signaling within vascular and cardiac tissues. By limiting angiotensin II (Ang II) generation and downstream AT_1_R signaling, ACE inhibitors reduce ROS-dependent NF-κB/AP-1 activation that drives MMP-9 transcription in vascular and cardiac cells [79]. Experimental and translational data show that ACE inhibition downregulates MMP-9 and related remodeling enzymes in aneurysm and myocardial-remodeling contexts: in a rat xenograft model of abdominal aortic aneurysm, perindopril attenuated aneurysmal degeneration in association with reduced remodeling enzyme activity; mechanistic work further demonstrates that ACE inhibitors can directly interact with MMP-9 and lower in vivo MMP-9 levels; and captopril suppresses left-ventricular inflammatory signaling with NF-κB inactivation, consistent with upstream control of MMP-9 expression [80,81,82]. Together with evidence that Ang II induces MMP-9 via NF-κB in vascular smooth muscle and immune cells, these findings support renin–angiotensin blockade as a strategy to recalibrate MMP-9 homeostasis in vascular walls, myocardium, and aneurysm models. Clinically, the evidence linking ACE inhibition to modulation of MMP-9 in human vascular disease is suggestive but still limited: small translational and animal-model studies (e.g., perindopril in aneurysm models) report reduced remodeling enzyme activity, yet large-scale trials of ACE inhibitors in aneurysm progression or myocardial remodeling have not been designed with MMP-9 suppression as a primary endpoint [83]. Thus, while mechanistic and preclinical signals remain consistent, the impact of ACE inhibitors on MMP-9 in human pathology must still be confirmed in focused trials with biomarker stratification and longitudinal assessment.

### 4.3. ARBs: Receptor-Level Modulation and Tissue Protection

Angiotensin II receptor blockers (ARBs) such as losartan, telmisartan, and valsartan attenuate MMP-9 induction by antagonizing angiotensin II type 1 receptor (AT_1_R) signaling in vascular smooth muscle cells, endothelial cells, and myocardium. Both in vitro and in vivo studies demonstrate that ARBs suppress MMP-9 mRNA expression and enzymatic activity across models of vascular inflammation and remodeling. For example, losartan abrogated Ang II-induced MMP-9 expression in rat atherosclerotic lesions and macrophage cultures, while telmisartan reduced MMP-9 secretion in activated macrophages [84]. In pulmonary hypertension models, co-treatment with losartan or captopril attenuated MMP-9 upregulation in lung tissue [85]. Beyond the vasculature, valsartan has been shown to inhibit MMP-9-dependent migration and invasion in tumor and fibrotic contexts [86]. Collectively, these data establish ARBs as targeted modulators that restrain pathological matrix MMP-9 activation while preserving the basal proteolytic activity required for vascular and tissue homeostasis. Translating these mechanisms in vivo, ARB therapy consistently reduces MMP-9 expression and activity in plasma and target organs, contributing to improved vascular stability and tissue remodeling. In experimental aneurysm models, AT_1_R blockade suppresses macrophage infiltration, elastin degradation, and aortic expansion in parallel with marked downregulation of *Mmp-9* transcripts within the vessel wall [87]. In hypertensive and post-infarction myocardium, chronic ARB administration limits collagen turnover and interstitial fibrosis, accompanied by reduced myocardial MMP-9 activity and inflammatory signaling [88]. Beyond the cardiovascular system, agents such as telmisartan and candesartan normalize circulating and renal MMP-9 levels in diabetic nephropathy, restoring endothelial and basement membrane integrity [89].

Together, these findings identify ARBs as clinically validated, upstream modulators of MMP-9. Through selective interruption of the Ang II–AT_1_R signaling axis, ARBs recalibrate vascular and tissue homeostasis while attenuating maladaptive, MMP-9-driven remodeling. By integrating anti-inflammatory, antioxidant, and anti-proteolytic mechanisms, ARBs exemplify context-specific, partial modulation of MMP-9-preserving physiological ECM dynamics across vascular, cardiac, and renal tissues while minimizing the liabilities associated with direct enzymatic inhibition.

### 4.4. Metabolic Modulators as Indirect Regulators of MMP-9: Linking AMPK and PPARγ Signaling to Extracellular Proteolysis

Beyond cardiovascular therapeutics, accumulating data indicate that metabolic modulators, notably metformin and pioglitazone, exert protective effects on MMP-9-driven inflammation and remodeling, linking AMP-activated protein kinase (AMPK) and peroxisome proliferator-activated receptor-γ (PPARγ) signaling to precise control of extracellular proteolysis across vascular and immune contexts [90]. Metformin lowers MMP-9 via AMPK-dependent suppression of NF-κB/AP-1 transcriptional programs and redox signaling; mechanistic reports further suggest direct interaction with MMP-9 leading to enhanced enzyme turnover, with concordant reductions in plaque and circulating MMP-9 and improved atherosclerotic stability in ApoE ^−^/^−^ mice [68]. Contemporary syntheses identify an AMPK-mTOR and oxidative-stress axis converging on MMP-9 transcription and release. Pioglitazone (PPARγ agonist) attenuates MMP-9 expression and activity across vascular and metabolic settings: in human saphenous-vein tissue from patients with diabetes, pioglitazone reduced MMP-dependent remodeling and improved ex vivo vascular reactivity; experimental and clinical studies in metabolic disease similarly report lower serum or tissue MMP-9, consistent with PPARγ-mediated dampening of NF-κB/AP-1 and macrophage MMP-9 release [91,92]. Taken together, metformin and pioglitazone function as mechanistically complementary, indirect modulators that partially suppress pathological MMP-9 while preserving physiological proteolysis—a profile aligned with the state-specific therapeutic window outlined above.

Clinically, randomized, MMP-9-focused evidence for antidiabetic agents remains limited; nevertheless, convergent mechanistic and translational data support a plausible class effect. SGLT2 inhibitors (e.g., empagliflozin) suppress MMPs activation and confer matrix-protective effects in preclinical vascular and cardiac models, and human studies consistently show cardiorenal benefit compatible with reduced protease-inflammation signaling [93]. Emerging human data also suggest that GLP-1 receptor agonists may improve endothelial function while lowering MMP-9 in select metabolic settings (e.g., polycystic ovary syndrome), though current reports are small and disease-specific, underscoring the need for targeted trials in diabetes and cardiovascular cohorts [94].

## 5. Anti-Inflammatory and Respiratory Modulators at the Immune-Epithelial Interface

Building on the cardiometabolic framework, recent focus has shifted toward how anti-inflammatory and respiratory therapeutics modulate MMP-9 through effects on immune regulation and oxidative stress. Whereas statins, angiotensin II receptor blockers, and metabolic agents primarily recalibrate vascular and metabolic homeostasis, corticosteroids, macrolides, and leukotriene receptor antagonists act at the immune–epithelial interface, where MMP-9 contributes to airway remodeling, neutrophil activation, and epithelial barrier disruption. The following section describes how these agents reshape MMP-9 dynamics across inflammatory and pulmonary beads, extending the principle of disease-specific, partial protease modulation beyond the cardiovascular system.

### 5.1. Glucocorticoids: GR-Mediated Repression of NF-κB/AP-1

Glucocorticoids (GCs) such as dexamethasone and prednisone suppress MMP-9 production primarily through glucocorticoid receptor (GR)-mediated repression of NF-κB and AP-1-dependent transcription in myeloid and stromal cells, thereby reducing neutrophil- and macrophage-derived MMP-9 at sites of inflammation. Translational data demonstrate that dexamethasone decreases both tissue and circulating MMP-9 in vivo while concurrently dampening NF-κB and MAPK (JNK/ERK) signaling—consistent with GR-driven transrepression of pro-inflammatory gene programs [95]. Recent mechanistic and clinical reviews further confirm that GCs inhibit NF-κB and AP-1 activity across pulmonary and systemic inflammatory systems, providing a unified molecular pathway for reduced MMP-9 transcription and secretion [96]. In human airway-relevant systems, corticosteroids suppress macrophage MMP-9 release (together with CXCL8 and TNF-α) in both healthy individuals and smokers, though this effect is attenuated in glucocorticoid-refractory diseases such as chronic obstructive pulmonary disease, underscoring both the on-target nature of the response and its dependence on disease type [97]. Foundational molecular studies further confirmed these findings, showing that liganded GR directly interferes with NF-κB/AP-1 transcriptional complexes, the canonical drivers of MMP-9 expression [98].

### 5.2. Paradoxical Effects of Chronic Glucocorticoid Exposure

Although glucocorticoids are widely used to suppress MMP-9 expression through NF-κB and AP-1 repression, several studies, particularly under chronic exposure or within specific tissue microenvironments, report paradoxical induction of MMP-9. In bone and osteoclastogenesis models, prolonged glucocorticoid treatment enhances osteoclast-derived MMP-9 activity, thereby promoting bone resorption and contributing to glucocorticoid-induced osteoporosis—a recognized adverse outcome of long-term steroid therapy [99]. Recent analyses of ECM regulation by GCs further highlight this duality: glucocorticoids can differentially modulate ECM turnover across tissues, shifting toward fibrotic or proteolytic remodeling depending on the local cytokine *milieu*, cellular composition, and duration of exposure [100]. Collectively, these findings underscore that glucocorticoid control of MMP-9 is not uniformly suppressive; rather, its direction and magnitude are case-dependent, shaped by tissue environment and treatment kinetics. Caution is therefore needed in extrapolating glucocorticoid therapy as a universally safe strategy for MMP-9 suppression, particularly in bone, chronic inflammatory, or long-term dosing regimen.

### 5.3. PDE4 Inhibitors: cAMP-Mediated Neutrophil Control

Phosphodiesterase-4 (PDE4) inhibitors, such as roflumilast, elevate intracellular cyclic Adenosine Monophosphate (cAMP) in neutrophils and macrophages, suppressing activation and degranulation and thereby reducing MMP-9 release. Early mechanistic studies in human neutrophils have shown that selective PDE4 inhibition significantly attenuates MMP-9, myeloperoxidase, and neutrophil elastase secretion in response to pro-inflammatory stimuli (e.g., fMLP combined with TNF-α), an effect not shared by PDE3 inhibitors or non-selective PDE inhibitors [101]. In more recent experimental settings, roflumilast significantly reduced MMP-9 levels in lipopolysaccharide-induced lung injury, reinforcing its modulatory role in inflammatory alveolitis [102]. While human COPD trials have not universally confirmed MMP-9 suppression (e.g., studies showing no change in plasma MMP-9 despite therapy), the convergence of mechanistic and disease-model data supports a role for PDE4 inhibitors in dampening MMP-9-driven tissue injury within respiratory and inflammatory settings.

In line with their cAMP-raising, anti-neutrophil mechanism, PDE4 inhibitors have shown signals-but not yet consistent, robust proof-of MMP-9 suppression in patients. A small prospective COPD study reported numerical decreases in serum MMP-9 with roflumilast alongside clinical improvement (CAT score), although changes did not reach statistical significance-underscoring limited power and substantial biomarker variability [103]. Larger COPD programs have focused on clinical outcomes (exacerbations, lung function) rather than MMP-axis biomarkers, and registered roflumilast trials rarely prespecify MMP-9 as an endpoint, making definitive conclusions difficult [104]. Contemporary reviews echo this gap: while PDE4 inhibition is mechanistically expected to dampen MMP-9 release from neutrophils/macrophages, human biomarker readouts remain inconsistent and need well-powered, biomarker-driven trials [105].

### 5.4. Macrolides: Transcriptional Suppression at the Epithelial Barrier

Macrolides such as azithromycin and clarithromycin exert host-directed, anti-inflammatory effects that are independent of antimicrobial action, consistently dampening airway epithelial and neutrophil/macrophage MMP-9 output in clinical and translational settings. Macrolides such as azithromycin and clarithromycin inhibit NF-κB and AP-1 transcriptional activity in epithelial and myeloid cells, thereby downregulating *Mmp-9* gene expression and reducing pro-MMP-9 protein output. For example, azithromycin has been shown to suppress NF-κB binding to DNA in airway models, diminishing MMP-9 promoter activation and cytokine-driven upregulation [106]. Clarithromycin similarly interferes with inflammatory cytokine induction (e.g., IL-1β, TNF) and downstream MMP-9 secretion in macrophages and vascular tissues, particularly in settings of endothelial activation or injury. In a randomized, double-blind trial of infants hospitalized with severe RSV bronchiolitis, azithromycin lowered upper-airway MMP-9 at the end of treatment, reinforcing a direct, short-term effect on protease tone in vivo [107]. Earlier phase-2 data in critically ill children with RSV respiratory failure likewise showed that high-dose azithromycin reduced endotracheal active and total MMP-9 within 72 h and was associated with shorter hospitalization, supporting a dose–responsive modulation of MMP-9 at the epithelial-neutrophil interface [108]. In chronic airway disease, a post hoc analysis of the BAT bronchiectasis trial found that while long-term azithromycin did not uniformly suppress sputum inflammatory markers at steady state, it attenuated the exacerbation-associated surge in MMP-9 relative to placebo—an effect consistent with macrolides mitigating neutrophil-driven proteolysis during inflammatory flares [109]. Clinically, these molecular effects translate into measurable benefits in lung transplantation, where azithromycin therapy lowers airway and bronchoalveolar MMP-9 levels, correlating with reduced bronchiolitis obliterans syndrome (BOS) incidence and improved graft stability. This finding supports the view that macrolides extend beyond anti-infective prophylaxis to act as immune–epithelial modulators, mitigating neutrophil-driven proteolysis and matrix injury that underlie chronic allograft dysfunction.

### 5.5. Leukotriene Antagonists: CysLT_1_ Blockade and Protease Regulation

By antagonizing CysLT_1_ signaling, montelukast down-tunes upstream NF-κB/AP-1 and oxidative pathways that drive *Mmp-9* transcription and leukocyte degranulation, thereby reducing neutrophil/macrophage MMP-9 release at inflamed interfaces in the airways and vasculature [110]. Recent preclinical data extend this mechanism across disease settings: in an abdominal aortic aneurysm model, montelukast curtailed aneurysm growth while downregulating MMP-9 (and MMP-2) in the aortic wall via AMPK-mTOR pathway effects, aligning MMP-9 suppression with structural protection of the vessel [111,112]. In neuroinflammation/ischemia, contemporary work shows montelukast re-programs microglial activation and improves post-stroke outcomes, a mechanistic context in which MMP-9 is a recognized effector of BBB injury; supporting biological plausibility for indirect MMP-9 attenuation in brain injury models, even when MMP-9 was not a prespecified endpoint [113]. Finally, cell- and tissue-level studies continue to document suppression of MMP-axis signaling by montelukast and related leukotriene antagonists, reinforcing a class effect on extracellular proteolysis beyond antimicrobial or bronchodilatory actions [114]. Montelukast induced inhibition of CysLT_1_ signaling offers a plausible, upstream route to indirectly attenuate MMP-9 (via dampening NF-κB/AP-1, oxidative stress, and neutrophil/macrophage degranulation). However, current evidence for on-treatment MMP-9 suppression in humans is sparse and proof-limited; most signals are preclinical (airway, vascular, brain-injury models) with heterogeneous endpoints and without standardized MMP-9 readouts. Thus, montelukast should be viewed as a context-dependent adjunct rather than a primary MMP-9-directed therapy.

### 5.6. NSAIDs: COX-2–PGE_2_–MMP-9 Axis Interference

Selective and non-selective NSAIDs attenuate COX-2-derived prostaglandin E_2_ (PGE_2_) signaling, thereby reducing PGE_2_-dependent induction of MMP-9 and limiting downstream proteolysis in inflamed tissues. Mechanistically, PGE_2_ engages EP receptors to upregulate MMP-9 via c-Src/JAK2/ERK to STAT3 and NF-κB/AP-1 pathways [115]; this axis is suppressed by celecoxib, which blocks bradykinin-triggered MMP-9 expression in astrocyte models and exemplifies prostaglandin-linked control of *Mmp-9* transcription. In musculoskeletal inflammation, recent work in human articular systems shows celecoxib downregulates MMPs through inhibition of NF-κB/JNK, aligning with COX-2 pathway blockade and supporting a chondroprotective effect. Macrophage-focused data further connect IL-6/COX-2/PGE_2_ signaling with MMP-9 expression, with celecoxib treatment reducing macrophage MMP-9 levels in fibrosis-related contexts [116,117]. Together, these findings substantiate a COX-2-PGE_2_-MMP-9 link across cancer, arthritis, and myeloid models and highlight NSAIDs potential to mitigate tissue destruction by halting prostaglandin-driven gelatinase activity. NSAIDs, especially COX-2 inhibitors like celecoxib, offer a mechanistically rational, readily available adjunct approach to moderate MMP-9 activity by interrupting the PGE_2_-mediated signaling cascade that amplifies protease transcription in inflamed tissues. Their potential lies in attenuating prostaglandin-driven MMP-9 induction in conditions like arthritis, cancer, or vascular inflammation, thereby limiting tissue degradation without directly targeting the catalytic site. However, the modulatory effect is likely modest and microenvironment-dependent, constrained by issues of dosing, specificity, and off-target NSAID toxicity, meaning that NSAIDs may best serve as complementary agents rather than primary MMP-9 inhibitors.

### 5.7. Direct Catalytic Modulation by Tetracyclines

In contrast to upstream immunomodulators, tetracyclines directly engage the metalloproteinase catalytic machinery while secondarily repressing pro-inflammatory transcription. Doxycycline and minocycline chelate active-site Zn^2+^ (and coordinating Ca^2+^) within the MMP catalytic domain, producing immediate, active-site level inhibition of MMP-9; in parallel, they suppress NF-κB/AP-1 signaling and can increase TIMP expression, thereby reducing *Mmp-9* transcription and net gelatinase output from myeloid and stromal cells. Recent clinical–translational data show that doxycycline treatment over 12 weeks lowers circulating MMP-9 (and MMP-2) with a reciprocal rise in TIMP-2 in patients, consistent with combined catalytic and transcriptional control in vivo. Complementing these observations, contemporary reviews and experimental studies affirm Zn^2+^ chelation as the principal catalytic mechanism and document minocycline-linked reductions in MMP-9 in neuroinflammatory injury models, aligning protease attenuation with improved barrier integrity and tissue outcomes [118,119,120]. Building on the mechanistic foundation of tetracyclines, sub-antimicrobial dose doxycycline (20 mg twice daily) represents the most clinically validated approach to sustained MMP-9 modulation. At this dose, doxycycline achieves selective inhibition of host MMPs without altering the commensal microbiota or exerting antibacterial pressure, enabling safe, chronic administration. Clinical trials in periodontitis, rosacea, and vascular inflammation consistently demonstrate reduced gingival crevicular fluid or plasma MMP-9, improved tissue stability, and lower inflammatory indices when sub-antimicrobial dose doxycycline (SDD) is added to standard care. Recent randomized studies and meta-analyses confirm durable reductions in MMP-9 activity and systemic inflammation markers (e.g., hs-CRP) during multi-month or multi-year therapy, underscoring its translational relevance as a host-response modulator rather than a conventional antibiotic. Given its well-established safety and regulatory approval for long-term use, SDD remains the prototype of selective, low-intensity MMP-9 inhibition applicable across chronic inflammatory disorders [121].

Despite this broad mechanistic spectrum, clinical translation of full-dose tetracyclines as MMP-9 inhibitors remains constrained by pharmacokinetic and safety limitations. Effective MMP inhibition often requires micromolar tissue concentrations, which overlap with ranges producing mitochondrial dysfunction, photosensitivity, and gut microbiome disturbance. Chronic exposure also raises antimicrobial-resistance concerns, especially in long-term inflammatory or vascular prevention settings. Recent pharmacodynamic studies confirm that plasma levels achieved with conventional antimicrobial dosing frequently exceed those needed for MMP inhibition, emphasizing the narrow therapeutic window. However, SDD (20 mg twice daily) circumvents these issues—sustaining MMP-9 suppression via non-bactericidal mechanisms, with an FDA-approved indication for chronic use in periodontitis and rosacea and a long-standing record of microbiologic safety and systemic tolerability. Thus, while broad clinical repurposing requires target-specific validation, low-dose tetracycline therapy represents the safest and most rational implementation of MMP-9 modulation to date.

## 6. Hormone-Related Modulation of MMP-9

### 6.1. mTOR Inhibition (Rapamycin/Sirolimus)

By inhibiting mTORC1, rapamycin (sirolimus) down-modulates pro-inflammatory transcriptional programs (notably NF-κB/AP-1) and promotes autophagy, thereby suppressing cytokine-induced *Mmp-9* transcription and reducing net gelatinolytic activity in relevant stromal and myeloid compartments. Contemporary mechanistic studies show rapamycin lowering MMP-9 expression in inflamed tissues and cell systems, for example, inflammatory chondrocytes exhibit rapamycin-mediated repression of MMP-9 via NF-κB inhibition coupled to autophagy induction; similar mTOR-centric control of matrix remodeling has been described in vascular wall biology [122]. Translational data across organ systems align with this mechanism: in atherosclerosis, nanoparticle co-delivery of rapamycin reduced plaque MMP-9 (and MMP-2) alongside improved endothelial function and plaque regression in ApoE^−^/^−^ mice; in breast ductal carcinoma in situ (DCIS), short-course everolimus (an mTOR inhibitor) decreased MMP-9 in tumor tissue and inhibited invasive progression in preclinical and patient-adjacent analyses; and in fibrotic/immune remodeling, sirolimus suppresses profibrotic stromal programs and circulating fibrocytes in human interstitial lung disease, a setting where MMP-9–linked matrix turnover contributes to pathology. Collectively, these studies support mTOR blockade as a means to lower MMP-9-driven remodeling in vascular, neoplastic, and fibrotic contexts, while highlighting the need for biomarker-anchored trials that prespecify MMP-9 endpoints in renal and transplant populations [123,124,125]. Direct human evidence linking rapamycin/sirolimus to on-treatment decreases in MMP-9 in renal or transplant cohorts is still limited; future studies should incorporate prespecified MMP-9 measurements (plasma/tissue/gelatinolytic activity) to define effect size and therapeutic windows in these settings.

### 6.2. MMP-9 and Estrogen and Selective Modulators (Estradiol, Tamoxifen)

Across vascular and immune interfaces, 17β-estradiol (E2) acting through estrogen receptors (ERα/ERβ) can reduce *Mmp-9* transcription by mitigating upstream inflammatory programs (NF-κB/AP-1) and related miRNA circuits in endothelial and stromal cells. In vivo E2 replacement in ovariectomized, high-fat-fed mice reduced vascular/metaflammatory (chronic metabolic inflammation) MMP-9 attributed to smooth-muscle and macrophage sources, consistent with ER-mediated anti-inflammatory signaling. At the same time, tumor-specific co-regulators can invert this effect: in ER-positive breast-cancer models, E2 induces MMP-9 via PELP1 to PI3K/Akt non-genomic signaling, underscoring tissue- and cofactor-dependent directionality [126,127]. Mechanistic and translational data together support a model in which estrogen’s vascular protection, improved endothelial function, tempered inflammation, and stabilized ECM, may partly reflect attenuation of MMP-9-driven remodeling in arterial beds. However, direct human trials with prespecified MMP-9 endpoints remain scarce in 2020–2025; future studies should integrate ER-pathway readouts (e.g., NF-κB/miRNA signatures) with circulating or tissue MMP-9 activity to quantify this contribution to atheroprotection [128].

In contrast to estradiol’s generally suppressive effects on MMP-9 in vascular settings, tamoxifen (and its active metabolites, e.g., 4-hydroxytamoxifen/endoxifen) can, in a subset of ER^+^ breast-cancer models, promote an invasive, matrix-degradative phenotype characterized by higher MMP-9 activity. Recent work shows that exposure to tamoxifen metabolites can select ER^+^ variants with marked MMP-9-mediated collagen degradation, a state that coincides with phenotypic drift toward endocrine resistance and non-genomic signaling adaptations [129,130]. More broadly, contemporary syntheses of the MMP field in breast cancer and chemoresistance emphasize that MMP network activation (including MMP-9) is enriched in aggressive or antiestrogen-resistant states—even if the specific MMP driver varies by model—underscoring a plausible mechanistic link between tamoxifen pressure and protease-enabled invasion [131]. Clinically, elevated tumor MMP-9 associates with poorer outcomes in early-stage disease, aligning with the concern that tamoxifen-selected phenotypes with higher MMP-9 activity could contribute to adverse biology in a subset of patients [132]. The bidirectional effects of estradiol and tamoxifen on *Mmp-9* expression underscore the contextual complexity of hormone–protease interactions, which depend on estrogen receptor (ER) subtype composition, cofactor availability, and the surrounding cellular *milieu*. Recent receptor-resolved studies demonstrate that ERα and ERβ elicit divergent transcriptional outcomes on MMP-associated genes, while GPER1 activation can modulate MMP-9 through non-genomic, cAMP- and PI3K-linked cascades. In vascular and immune cells, these distinctions explain why estradiol typically suppresses MMP-9 under anti-inflammatory ERβ dominance, whereas in certain neoplastic contexts dominated by ERα or GPER1 signaling, estrogen or selective modulators like tamoxifen may instead enhance MMP-9 expression and activity. Moreover, the tissue microenvironment—including cytokine gradients, hypoxia, and matrix stiffness—further shapes ER co-regulator recruitment, adding another regulatory tier to MMP-9 control [133]. Collectively, these data highlight that estrogenic modulation of MMP-9 is receptor- and environment-specific rather than uniformly stimulatory or suppressive, a principle that carries implications for tailoring hormonal and anti-estrogen therapies in both vascular and oncologic disease states.

### 6.3. MMP-9 at the Crosslink with Progesterone and Androgen Pathways

Emerging evidence indicates that progesterone (P4) and related steroid hormones modulate *Mmp-9* expression and activity at the maternal–fetal interface, with important implications for implantation, placentation, and the maintenance of pregnancy. In human decidual and placental models, P4 attenuates infection- or cytokine-driven pro-MMP-9 induction (e.g., *E. coli* stimulation of decidual cells) and reduces type IV collagen degradation in vitro, signifying a protective, remodeling-modulating role [134]. Clinical observational studies bolster this notion: elevated MMP-9 levels in vaginal secretions or cervical tissue correlate with increased risk of preterm birth and spontaneous labor, suggesting dysregulation of the steroid-hormone-MMP-9 axis may underlie pathological remodeling [135]. Importantly, the hormonal regulation of MMP-9 appears context- and tissue-specific, in the endometrium, P4 receptor activation suppresses MMP-9 during the secretory phase to maintain stromal integrity and prevent premature breakdown of the ECM, whereas in states of progesterone resistance (e.g., preterm labor, placenta accreta) enhanced MMP-9 expression is frequently observed. Together, these findings underscore the concept that progesterone-mediated control of MMP-9 is central to reproductive tissue homeostasis, and that perturbations in this balance may contribute to adverse pregnancy outcomes through uncontrolled matrix degradation.

Emerging preclinical and translational studies suggest that androgens and androgen-receptor (AR) signaling may influence MMP-9 expression and ECM remodeling in prostate and vascular tissues. In prostate cancer fibroblast and tumor microenvironment models, AR activation has been linked to upregulation of MMP-9/VEGF signaling via PIP5K1α/AKT crosstalk, implicating a molecular axis by which androgen signaling promotes proteolysis and vascular remodeling in tumor stroma [136]. Conversely, androgen deprivation therapy (ADT)—by suppressing AR-mediated transcription—may indirectly reduce MMP-9 activity, contributing to ECM stabilization; this concept aligns with clinical observations that ADT is associated with structural vascular changes and altered remodeling in patients [137]. However, ADT is also linked to cardiovascular side effects (e.g., increased heart failure risk), which could paradoxically enhance remodeling stress and offset potential MMP-9 suppression benefits in the vasculature [138]. As of now, direct human data measuring on-treatment MMP-9 levels in response to ADT are sparse. Targeted biomarker studies are needed to clarify whether MMP-9 is a modifiable mediator of ADT’s tissue remodeling effects—especially in prostate cancer progression, vascular injury, and cardiac remodeling. Hormone-related modulation of MMP-9 is highly case-dependent, reflecting the intricate interplay between receptor subtype, local microenvironment, and disease state. Estradiol and progesterone generally exert protective, anti-proteolytic effects in vascular and reproductive tissues by suppressing NF-κB/AP-1 signaling and stabilizing ECM integrity. In contrast, tamoxifen and, in some settings, testosterone or its analogs can enhance MMP-9 activity, promoting matrix degradation, invasion, or adverse remodeling—particularly in hormone-sensitive cancers. Collectively, these findings underscore that hormone-MMP-9 interactions are tissue- and receptor-specific, necessitating precision therapeutic approaches that account for hormonal context, receptor isoform dominance, and disease-specific signaling networks.

## 7. Nutritional and Microbiome-Linked Modulation

### 7.1. Omega-3 Fatty Acids and Antioxidant Vitamins

Contemporary work supports that EPA/DHA attenuate *Mmp-9* transcription and secretion primarily by mitigating NF-κB/AP-1-driven inflammatory signaling in myeloid and endothelial compartments; mechanistic summaries explicitly note MMP-9 modulation in conjunction with barrier and immune effects [139]. In human inflammatory disorders, omega-3s used as adjunct therapy improve clinical and biomarker profiles; in periodontitis, recent trials and syntheses report better healing and inflammatory readouts with supplementation alongside standard care, with MMP-9 outcomes variably included but directionally favorable in gingival fluids or local tissue [140,141]. In atherosclerosis models, high-dose omega-3 regimens stabilize plaques and blunt inflammatory remodeling; findings consistent with a reduction in protease-driven matrix degradation (with MMP-9 a recognized contributor to plaque vulnerability). While direct on-treatment human MMP-9 data remain limited, these preclinical and mechanistic signals support a biologically plausible link between omega-3s, lowered NF-κB tone, and reduced gelatinase activity in the vascular wall [142].

### 7.2. Micronutrient and Dietary Pattern Effects

Across inflammatory disorders, micronutrients can indirectly regulate MMP-9 by impacting upstream immune-redox signaling. Vitamin D engages VDR-dependent transcriptional control to repress NF-κB/AP-1 programs; clinically, a recent supplementation study demonstrated significant decreases in gingival crevicular fluid MMP-9 after one month, supporting on-treatment protease reduction in humans [143]. Antioxidant vitamins (notably vitamin C) mitigate ROS-driven induction of MMP-9; in a prospective ICU cohort, ascorbic acid (with/without thiamine) lowered circulating MMP-9, consistent with redox-sensitive control of gelatinase expression [144]. Finally, B-vitamin/folate strategies address homocysteine, a vascular toxin that upregulates MMP-9 and disrupts endothelial integrity; mechanistic human data link hyperhomocysteinemia to excess MMP-9, providing a biologic rationale for homocysteine-lowering regimens as indirect MMP-9 modulators in atherosclerotic risk states [145].

Adherence to a Mediterranean-style dietary pattern (rich in fruits, vegetables, legumes, whole grains, olive oil, fish, and wine in moderation) aligns with lower systemic inflammatory tone, and several contemporary syntheses attribute part of this signal to polyphenol-driven repression of NF-κB/AP-1 programs that regulate MMP-9 function. Although direct, on-diet human trials with prespecified MMP-9 endpoints remain elusive, recent reviews of Mediterranean-diet polyphenols and antioxidant compounds document preclinical suppression of MMP-9 expression and activity, providing a biologically credible link between diet quality and protease attenuation [146,147]. In contrast, the evidence for energy reduction and weight loss on MMP-9 is more solid: clinical weight-loss interventions, particularly bariatric surgery cohorts, report significant decreases in circulating MMP-9 over 3 to 12 months that track with BMI reduction, consistent with relief of adipose-derived inflammatory signaling (e.g., TNF-α/IL-6) upstream of MMP-9. Moreover, a randomized trial found that improving vitamin D status during a calorie-restricted weight-loss program augmented reductions in MMP-9, suggesting that micronutrient repletion can potentiate diet-induced protease down-modulation. Together, these data support weight loss as a robust, clinically actionable lever to dampen MMP-9 activity, while Mediterranean-pattern eating supplies a mechanistically coherent, polyphenol-rich framework likely to reinforce this effect [148]. Mediterranean-style, polyphenol-rich eating is mechanistically aligned with lower MMP-9 drive, while caloric restriction/weight loss yields measurable human declines in circulating MMP-9—together supporting diet as a low-toxicity adjunct for context-specific, partial modulation of the MMP-9 axis (Table 1).

### 7.3. Microbiome and Gut-Systemic MMP-9 Axis

Emerging data link gut dysbiosis with systemic upregulation of MMP-9, mechanistically via barrier disruption and LPS/TLR–NF-κB/AP-1 activation that drives *Mmp-9* transcription and neutrophil/macrophage degranulation. Recent syntheses explicitly place MMP-9 within the gut–inflammation axis that propagates injury beyond the intestine (gut–liver/lung/vascular), strengthening biological plausibility for microbiome-targeted modulation [149]. Clinically, microbiome-directed therapy can reduce MMP-9 in select settings: in ulcerative colitis, mesalazine plus a bifidobacteria-based probiotic lowered fecal MMP-9 alongside improved microbial composition versus mesalazine alone, indicating that correcting dysbiosis can attenuate protease tone in vivo [150]. Translational respiratory data point out the same way—probiotic blends in COPD models decreased lung MMP-9 mRNA/protein and inflammatory cytokines, supporting a gut–lung axis in which probiotics/prebiotics dampen MMP-9-linked remodeling; human COPD trials increasingly show safety and anti-inflammatory signals, though standardized MMP-9 endpoints remain uncommon [151]. Dysbiosis plausibly amplifies systemic MMP-9, and early interventional evidence suggests that probiotic/prebiotic strategies can partially reduce MMP-9 in disease-specific contexts; definitive human trials with prespecified MMP-9 (mass and activity) outcomes are the key next step.

## 8. Future Directions and Clinical Translation

The therapeutic landscape for MMP-9 modulation is shifting from indiscriminate inhibition toward precision, context-dependent control of its proteolytic and signaling functions. Accumulating clinical and translational data emphasize that partial or tissue-specific suppression, rather than complete blockade, may maximize benefit while preserving physiologic remodeling and repair. Moving forward, a major research priority lies in the integration of MMP-9 as a dynamic biomarker—measurable in plasma, tissue, or extracellular vesicles—to stratify patients, monitor therapeutic response, and guide dose titration of repurposed agents such as statins, tetracyclines, or PPAR-γ agonists.

Future research should focus on translating this mechanistic understanding into precision pharmacology. Biomarker-focused clinical trials incorporating circulating, tissue, and extracellular-vesicle MMP-9 profiling are needed to define therapeutic windows and identify responder subgroups. Integration of multi-omics profiling with spatial transcriptomics could unravel tissue-specific signaling networks that govern MMP-9 activation, while structure-guided design of domain-selective inhibitors and antibody or TIMP-mimetic scaffolds may overcome the historical limitations of broad-spectrum metalloproteinase blockade. Finally, combination strategies coupling pharmacologic agents with nutritional or microbiome-directed interventions offer a promising route toward restoring matrix homeostasis. These advances collectively position MMP-9 as a measurable, druggable, and clinically actionable target in the next generation of anti-inflammatory and antifibrotic therapies. The authors would like to highlight critical points of interest:

Isoform- and activation-state-specific assays: Future studies must move beyond total MMP-9 quantification to distinguish pro-MMP-9, active MMP-9, and MMP-9 bound to inhibitors (e.g., TIMP-1). Such granularity will clarify when MMP-9 is truly proteolytically active in disease versus present as an stationary zymogen, thereby improving mechanistic interpretation and biomarker constancy.

Cell-type- and compartment-specific mapping: Given that MMP-9 is produced by diverse types of cells (neutrophils, macrophages, endothelial cells, pericytes), there is a pressing need for spatially resolved analyses (e.g., single-cell RNA-seq, in situ zymography) to reveal which cellular sources contribute to pathologic MMP-9 activities in each disease conditions. This approach will refine the translational targeting of MMP-9.

Proteome–substrate interaction networks: Future work should integrate degradomic approaches (e.g., TAILS, terminal-amide isotopic labeling) to identify the full complement of in vivo MMP-9 substrates in disease compartments. By defining substrate repertoires, we can better link MMP-9 activity to downstream functional consequences (e.g., barrier disruption, growth-factor release) and identify novel therapeutic nodes.

Disease-specific inhibition strategies: The ubiquitous physiological roles of MMP-9 demand that inhibition be precisely targeted. Future preclinical studies should assess temporal (over the disease course) and spatial (which tissue/compartment) inhibition windows, perhaps using inducible genetic models or highly selective small-molecule or antibody inhibitors, to establish therapeutic windows that preserve beneficial functions while blocking deleterious effects.

Clinical–translational biomarker development: Longitudinal human studies should evaluate whether circulating or tissue-derived active MMP-9 (rather than total enzyme) predicts disease progression or response to therapy across fibrosis, vascular, and oncologic indications. Moreover, combining MMP-9 readouts with upstream modulators (e.g., PON1, homocysteine) may improve stratification of patients who will benefit most from MMP-9-directed interventions.

Integration with systems-biology frameworks: It is timely to introduce MMP-9 into systems-scale models of inflammation, ECM turnover, vascular and interstitial remodeling. Computational modeling of MMP-9 kinetics, substrate flux, and network interactions will enable hypothesis generation and optimization of intervention strategies across disease types.

From a pharmaceutic perspective, the successful translation of MMP-9-targeted interventions depend not only on molecular selectivity but also on spatiotemporal control of drug exposure within protease-active microenvironments. Advances in nanoparticle, liposomal, and hydrogel-based formulations now enable targeted or sustained delivery of MMP-9 modulators—ranging from tetracyclines and TIMP-mimetics to small-molecule zinc chelators—directly to inflamed, fibrotic, or tumor tissues while minimizing systemic toxicity. Local delivery approaches (e.g., inhaled corticosteroids and PDE4 inhibitors in pulmonary inflammation, or topical and intralesional formulations for wound and vascular repair) illustrate the feasibility of achieving highly specific protease modulation at clinically relevant concentrations. Emerging responsive platforms; including pH-, redox-, and enzyme-triggered release systems, offer additional precision by coupling therapeutic release to the proteolytic *milieu* itself. Together, these formulation strategies bridge the gap between mechanistic insight and therapeutic implementation, situating MMP-9 modulation firmly within the translational pharmacology landscape.

Ultimately, advancing MMP-9-directed therapy will require prospective, biomarker-anchored clinical trials that define therapeutic windows, validate composite endpoints (e.g., MMP-9/TIMP-1 ratios), and identify responders across cardiovascular, neuroinflammatory, and fibrotic diseases. These approaches will move the field from conceptual modulation toward clinically actionable precision pharmacology, positioning MMP-9 as both a disease effector and a measurable therapeutic target in next-generation drug development.

## 9. Summary

Matrix metalloproteinase-9 (MMP-9) is increasingly recognized as a central mediator of tissue remodeling and chronic inflammation, yet therapeutic targeting remains challenging. This review synthesizes current evidence on the capacity of approved drugs and adjunctive interventions to modulate MMP-9 activity across cardiovascular, respiratory, endocrine, and inflammatory diseases. Cardiometabolic drugs, including statins, ACE inhibitors, ARBs, metformin, and pioglitazone, consistently suppress MMP-9, with translational implications for plaque stabilization, aneurysm control, and improved vascular outcomes. Anti-inflammatory and respiratory agents such as glucocorticoids, PDE4 inhibitors, macrolides, montelukast, and NSAIDs further attenuate airway and systemic inflammation by reducing MMP-9 release from neutrophils and macrophages. Tetracyclines, particularly sub-antimicrobial dose doxycycline, provide one of the most direct and clinically validated means of MMP-9 inhibition, supported by trials in periodontal disease and vascular remodeling. Hormone-related therapies, including estradiol, tamoxifen, and rapamycin, demonstrate context-dependent and sometimes opposing effects on MMP-9, underscoring the complexity of endocrine regulation. Nutritional strategies, notably omega-3 fatty acids and antioxidant vitamins, offer modest but reproducible suppression of MMP-9, making them attractive as adjunctive therapies.

While the amount of evidence highlights opportunities for repurposing existing therapies, the field remains limited by heterogeneous study designs, variable endpoints, and the lack of selective, pathology-sensitive inhibitors. Moreover, most data derive from preclinical or small clinical cohorts, and long-term outcomes linked specifically to MMP-9 modulation remain underexplored. Future research should prioritize biomarker-guided trials, mechanistic dissection of tissue-specific effects, and integrated strategies that combine pharmacologic and lifestyle interventions. Collectively, these approaches may enable safe, highly targeted modulation of MMP-9 and reinvigorate its potential as a therapeutic target.

## Figures and Tables

**Table 1:** Therapeutic and Adjunctive Agents Modulating MMP-9 Activity.

Drug/Class	Mechanistic Target	References	Clinical/Experimental Context	Effect On MMP-9
Statins	Rhoa/Rac1 NF-Κb/AP-1	74, 77	Atherosclerosis, Vascular Inflammation	(−) Expression/Activity
ACE Inhbs/Arbs	Ang II → AT_1_R	81, 83	Aneurysm, Cardiac Remodeling	(−) MMP-9 Expression
Metformin	AMPK/Mtor	91	Diabetes, Vascular Disease	(−) MMP-9 + (+) Stability
Pioglitazone	Pparγ	93	Metabolic Inflammation	(−) Activity
Doxycycline	Zn^2+^ Chelation/TIMP-2	122	Periodontitis, Vascular Remodeling	(−) Activity
Macrolides	NF-Κb/AP-1	108	Airway Inflammation	(−) Expression
Montelukast	Cyslt_1_/AMPK/Mtor	112	Aneurysm, Neuroinflammation	(−) Activity
Rapamycin	Mtorc1 Inhibition	125	Fibrosis, Transplant Models	(−) MMP-9 Expression
Omega-3/Vit. D	Nf-Κb/Redox Modulation	144	Inflammatory, Metabolic Disease	(−) Expression

## Data Availability

Not applicable.

## Abbreviations

ACE	Angiotensin-Converting Enzyme
ADT	Androgen Deprivation Therapy
ALI	Acute Lung Injury
Ang II	Angiotensin II
AP-1	Activator Protein-1
ARB	Angiotensin II Receptor Blocker
AR	Androgen Receptor
BBB	Blood–Brain Barrier
BMI	Body Mass Index
cAMP	Cyclic Adenosine Monophosphate
COX-2	Cyclooxygenase-2
DCIS	Ductal Carcinoma In Situ
DNA	Deoxyribonucleic Acid
DHA	Docosahexaenoic Acid
ECM	Extracellular Matrix
EPA	Eicosapentaenoic Acid
ER	Estrogen Receptor
ERα/ERβ	Estrogen Receptor Alpha/Beta
FDA	Food and Drug Administration
GPER1	G Protein-Coupled Estrogen Receptor 1
HFD	High-Fat Diet
ICU	Intensive Care Unit
IL-6	Interleukin-6
MMP-9	Matrix Metalloproteinase-9
MMPs	Matrix Metalloproteinases
mRNA	Messenger Ribonucleic Acid
mTOR	Mechanistic Target of Rapamycin
mTORC1	Mechanistic Target of Rapamycin Complex 1
NF-κB	Nuclear Factor Kappa-Light-Chain-Enhancer of Activated B Cells
NO	Nitric Oxide
PG	Prostaglandin
PI3K/Akt	Phosphoinositide 3-Kinase/Protein Kinase B Signaling Pathway
PK	Pharmacokinetics
PPAR-γ	Peroxisome Proliferator-Activated Receptor Gamma
PUFA	Polyunsaturated Fatty Acid
RAAS	Renin–Angiotensin–Aldosterone System
ROS	Reactive Oxygen Species
SDD	Sub-Antimicrobial Dose Doxycycline
TGF-β1	Transforming Growth Factor Beta-1
TIMP-1	Tissue Inhibitor of Metalloproteinases-1
TLR	Toll-Like Receptor
TNF-α	Tumor Necrosis Factor-Alpha
VDR	Vitamin D Receptor
VEGF	Vascular Endothelial Growth Factor
